# Reinfection after COVID vaccination - The challenge is not yet over

**DOI:** 10.12669/pjms.37.7.5228

**Published:** 2021

**Authors:** Shanila Ahmed, Noman Ali

**Affiliations:** 1 Shanila Ahmed, M.B.B.S. Resident, Department of Medical Oncology, Aga Khan University Hospital, Stadium Road, Karachi, Pakistan; 2 Noman Ali, M.B.B.S, FCPS (EM) Assistant Professor, Department of Emergency Medicine, Aga Khan University Hospital, Stadium Road, Karachi, Pakistan

COVID-19 has created a global public health emergency and was reported as pandemic in March 2020. Middle to low-income Asian countries were the worst affected. A lot has changed in the past few months about Covid pandemic due to the arrival of vaccine against coronavirus.

As per global vaccination data, less than 25% population of Pakistan has received vaccine till date and around 100 million people are fully vaccinated so far.^1^ In general, the vaccines for Covid are highly effective in preventing disease, with some even more than 90% effective.^2^ Nevertheless, post vaccination a small number of people can still get COVID-19 after exposure to Sars-Cov2. Such cases are considered to be “***vaccine breakthrough cases***.” However, people who have been vaccinated are less likely to develop severe symptoms.

Covid vaccine usually takes around two to three weeks for the body to build immunity. Vaccine’s actual effectiveness may vary depending upon the type of vaccine, one’s underlying immune reserves and exposure risk.

Initially when vaccination was started in Pakistan, a fair decline in number of new cases, inpatient admissions, and deaths due to Covid-19 was noted. But after May 2021 there was a reversal in downward trajectory of the cases. Currently our country is dealing with vaccine breakthrough cases due to B.1.617.2 (Delta) Variant. This variant found to be more contagious which is causing rapid increase in cases of Covid. Considering this fact, in July 2021 Centers for Disease Control and Prevention (CDC) issued updated guidelines. CDC reinforced the matter of urgently increasing COVID-19 vaccination coverage and also emphasized on recommendation of wearing a mask in public indoor places, even after full vaccination.

In Pakistan majority of the population has received vaccines that comprised of two doses. A study was conducted in Singapore to compare the clinical and biological features of delta variant between fully vaccinated and unvaccinated individuals, and they found that Covid vaccines are highly effective against this variant as well.^3^ However no updated data in Pakistan has been reported till the time of writing this communication regarding effectiveness of BBIBP-CorV (Sinopharm) and Sinovac vaccines against these variants.

In these difficult circumstances we really hope that soon we will be able to identify more variants of vaccine breakthrough cases that are specific to our population but if we fail to do so then probably we will have to face one after another wave that would be more disastrous and at that time, we might need different form of vaccine to protect our nation.
